# Valsartan impedes epinephrine-induced ICAM-4 activation on normal, sickle cell trait and sickle cell disease red blood cells

**DOI:** 10.1371/journal.pone.0216467

**Published:** 2019-05-13

**Authors:** Jing Zhang, Sasia-Marie Jones, George Lykotrafitis, Biree Andemariam

**Affiliations:** 1 Department of Mechanical Engineering, University of Connecticut, Storrs, Connecticut, United States of America; 2 New England Sickle Cell Institute, Division of Hematology-Oncology, Neag Comprehensive Cancer Center, UCONN Health, University of Connecticut, Farmington, Connecticut, United States of America; 3 Department of Biomedical Engineering, University of Connecticut, Storrs, Connecticut, United States of America; Max Delbruck Centrum fur Molekulare Medizin Berlin Buch, GERMANY

## Abstract

Abnormal red blood cell (RBC) adhesion to endothelial αvβ3 plays a crucial role in triggering vaso-occlusive episodes in sickle cell disease (SCD). It is known that epinephrine, a β-adrenergic receptor (β-AR) stimulator, increases the RBC surface density of active intercellular adhesion molecule-4 (ICAM-4) which binds to the endothelial αvβ3. It has also been demonstrated that in human embryonic kidney 293 cells, mouse cardiomyocytes, and COS-7 cell lines, the β-adrenergic and renin-angiotensin systems are interrelated and that there is a direct interaction and cross-regulation between β-AR and angiotensin II type 1 receptor (AT1R). Selective blockade of AT1R reciprocally inhibits the downstream signaling of β-ARs, similar to the inhibition observed in the presence of a β-AR-blocker. However, it is not known if this mechanism is active in human RBCs. Here, we studied the effect of valsartan, an AT1R blocker, on the surface density of active ICAM-4 receptors in normal, sickle cell trait, and homozygous sickle RBCs. We applied single molecule force spectroscopy to detect active ICAM-4 receptors on the RBC plasma membrane with and without the presence of valsartan and epinephrine. We found that epinephrine significantly increased whereas valsartan decreased their surface density. Importantly, we found that pretreatment of RBCs with valsartan significantly impeded the activation of ICAM-4 receptors induced by epinephrine. The observed reduced expression of active ICAM-4 receptors on the RBC plasma membrane leads us to conjecture that valsartan may be used as a supporting remedy for the prevention and treatment of vaso-occlusive crisis in SCD.

## Introduction

In sickle cell disease (SCD), an inherited β-globin gene point mutation encodes abnormal hemoglobin (HbS), which under deoxygenated conditions polymerizes to form long stiff filaments deforming red blood cells (RBCs) from a biconcave to a sickle shape [1–3]. Sickle cell trait (SCT) is a heterozygous state characterized by the presence of both normal hemoglobin (HbA) and HbS. While patients with SCD suffer increased mortality and often widespread chronic organ damage, individuals with SCT are generally asymptomatic and live a normal lifespan. However, under extreme conditions, such as hypoxia, acidosis, and dehydration, individuals with SCT can develop a syndrome resembling SCD with vaso-occlusive sequelae [4, 5].

It has been shown that homozygous sickle RBCs (SS-RBCs) are stiffer and more viscous than normal (wild-type, WT) RBCs [6–11]. In addition, SCT-RBCs and SCD-RBCs are more adhesive than WT-RBCs due to a higher activation of surface receptors [12–15]. Increased RBC adhesion, enhanced stiffness, and abnormal shape contribute to the entrapment of RBCs in the blood vessels and impediment of blood flow triggering painful vaso-occlusive episodes (VOEs), the hallmark of SCD [16].

**Fig 1:**
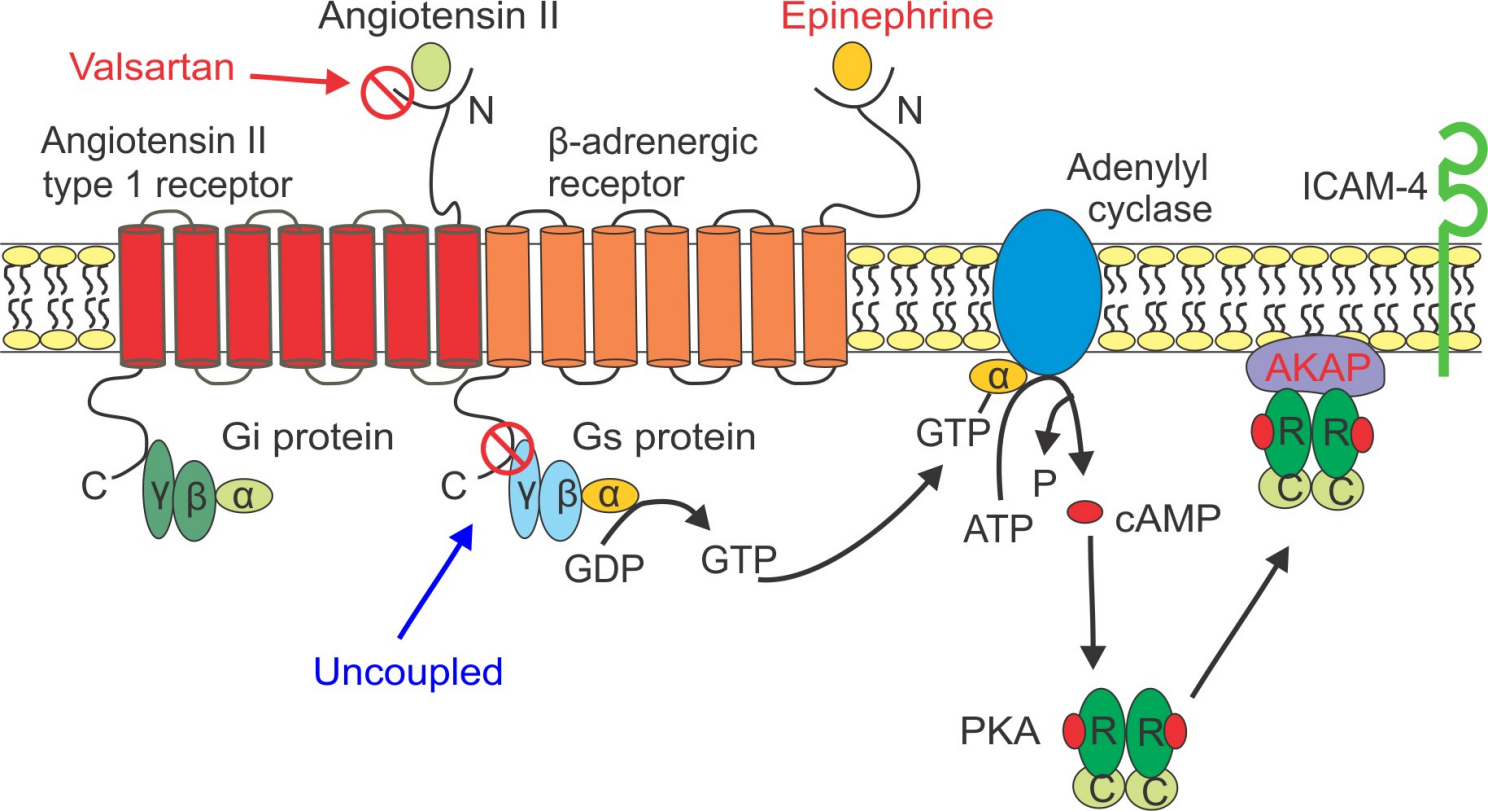
RBC cAMP-PKA-dependent pathway and AT1R vs β-AR dimer/complex.

Human RBCs express a large number of adhesion receptors on their membrane such as basal cell adhesion molecule/Lutheran (BCAM/Lu) and intercellular adhesion molecule-4 (ICAM-4), both of which are known to play a significant role in the genesis of vaso-occlusion in SCD [8, 14, 16–20]. BCAM/Lu and ICAM-4 bind with high affinity specifically to laminin-α-5, a component of the endothelial subcellular matrix, and to endothelial integrin αvβ3 respectively, and are the primary receptors that mediate the adhesion of SS-RBCs to endothelium. Surface receptors can be activated through certain intracellular signal pathways [8, 17, 18, 21, 22]. Previous research shows that BCAM/Lu and ICAM-4 receptor activation is mediated via the cyclic adenosine monophosphate-protein kinase A (cAMP-PKA) pathway [8, 14, 18, 23]. As shown in Fig 1, stimulation of β2-adrenergic receptors (β2-ARs) with an agonist, such as epinephrine, a catecholamine commonly secreted due to physical stress, can cause dissociation of the alpha subunit of its coupled Gs protein, which then activates adenylyl cyclase (AC) [21, 24–26]. AC catalyzes the conversion of adenosine triphosphate (ATP) to cAMP which then activates PKA [8, 27]. A kinase anchoring proteins (AKAPs) anchor PKA to specific intracellular sites on the RBC membrane where PKA can alter the phosphorylation state of neighboring RBC adhesion receptors thereby causing their activation [8, 23]. Propranolol, as a β-AR antagonist, has been shown to be effective in vivo both in mice and in humans to reduce epinephrine-stimulated SS-RBC adhesion and to prevent vaso-occlusion in mice [28]. These findings suggest that RBC receptor blockers may have a role as anti-adhesive therapy for SCD.

The adrenergic system and renin-angiotensin system are interrelated (Fig 1) [29]. Angiotensin II (Ang II) is an octapeptide hormone and neurotransmitter that plays a significant role in the regulation of renal, cardiovascular, and endocrine function [30]. Ang II exerts its biological effects through signaling pathways activated upon binding to two receptors, Ang II type 1 receptor (AT1R) and Ang II type 2 receptor (AT2R), and relying mainly on binding to AT1R [31]. AT1R, similarly to β2-AR, is a member of the seven-transmembrane-domain, the superfamily of G protein-coupled receptors (GPCRs), and it mediates most of the pathophysiological effects of Ang II, including vasoconstriction, inflammation, growth, and fibrosis. Ang II binding to the AT1R results in coupling of G proteins (Gq/G11and/or Gi/Go, G12, G13) to the C-terminus of the receptor and to stimulation of several intracellular/cytoplasmic signaling pathways [31–34]. Although receptors in the GPCR superfamily were initially believed to function as monomeric entities, mounting evidence suggests that they are oligomers and function either through interactions with identical units (homodimers) or with other GPCRs (heterodimers) [35–40]. In addition, many identified homodimers and heterodimers of GPCRs have different ligand binding [41, 42], signaling [43, 44] and trafficking [45] properties from their constituent monomers.

Valsartan is an AT1R antagonist developed clinically for the treatment of hypertension and congestive heart failure. It has been found that it reduces the risk of death after a myocardial infarction. Barki-Harrington et al [36] showed the existence of direct cross talk between AT1R and β-AR at the receptor level in human embryonic kidney (HEK) cells, mouse cardiomyocytes, and COS-7 cell lines. They found that a single receptor antagonist blocks downstream signaling of both AT1R and β-AR simultaneously. In addition, the ability of the antagonist to inhibit receptor signaling was primarily dependent on the expression levels of both receptors. Specifically, they showed that valsartan trans-inhibited β-ARs by functionally uncoupling β-AR from its associated Gs protein and interrupted its downstream signaling pathway. Further, their findings from differential epitope tagging and selective coimmunoprecipitation suggested that β2-AR and AT1R were assembled as oligomers on the plasma membrane (Fig 1). Thus, the trans-inhibition effect most likely worked on the β2-AR and AT1R constitutive complex formed on the cell membrane. However, it is not known if this cross-talk between β-AR and AT1R is active in RBCs.

In this study, we investigated the influence of valsartan on the cAMP-PKA pathway by measuring the surface expression of active ICAM-4 receptors on human WT-, SCT-, and SS-RBCs. We employed an atomic force microscopy (AFM) technique called single molecule force spectroscopy (SMFS) [8] which can detect single functional receptors on RBCs as well as measure the unbinding force between a receptor and its corresponding ligand [23, 46–51]. We demonstrated that pretreatment of RBCs with valsartan significantly hindered the activation of ICAM-4 receptors induced by epinephrine. The findings of this work suggest that administration of valsartan offers a possible way to mitigate vaso-occlusive episodes in SCD.

## Materials and methods

### Human subjects, blood collection and preparation

This study was approved by the Institutional Review Boards of the University of Connecticut-Storrs and UCONN-Health. All subjects were at least 18 years old and were ineligible to participate if they were pregnant, breastfeeding, or within 3 months postpartum; used anti-lipid therapy within the previous week; were taking an investigational drug; or received a blood transfusion within the previous 3 months. Additionally, homozygous SCD (SS) patients were only eligible to participate if they were at steady state. Steady state was defined as having not received a parenteral opioid in the previous 4 weeks, no pain greater than the usual daily level for 4 weeks, and no increase in usual non-parenteral opioid medication in the previous 4 weeks. Hydroxyurea (HU) treatment was an exclusion criterion because HU is known to inhibit BCAM/Lu adhesion to laminin [8, 52] and we inferred that HU might act in a similar way inhibiting activation of ICAM-4. Subject demographic and clinical characteristics are provided in Table 1.

**Table 1:** Subject demographic and clinical characteristics.

Factors	WT	SCT	SS
Subject numbers	5	3	6
Age, mean, SD	42 (14)	34 (5)	27 (5)
Gender, n, %	female 3 (60) male 2 (40)	female 3 (100)	male 5 (83) female 1 (17)
Race, n, %	African-American 4 (80) White 1 (20)	African-American 2 (67) Latino 1 (33)	African-American 6 (100)
Hemoglobin, g/dL, mean, SD	14 (1)	13 (1)	9 (1)
Leukocyte count, K/μL, mean, SD	7 (2)	6 (1)	12 (2)
Platelet, K/μL, mean, SD	222 (37)	228 (29)	430 (156)
Neutrophil, %, mean, SD	47 (15)	58 (10)	58 (10)
Reticulocytes, %, mean, SD	N/A	N/A	13 (4)
LDH, U/L, mean, SD	N/A	N/A	497 (82)

Blood samples were collected into heparinized tubes from eligible subjects after they provided written informed consent. Whole blood was centrifuged at 500 ×g for 5 min at 4°C to isolate the RBCs. The plasma and buffy coat were removed. The remaining RBCs were washed three times with Alsever’s solution and stored at 4°C for up to 7 days. During experiments, RBCs were seeded in a glass bottom petri dish coated with 1 mg/mL poly-L-lysine solution (Sigma-Aldrich, St. Louis, MO). At this concentration, the RBCs were immobilized while maintaining their original biconcave shape. All experiments were performed under normoxic conditions. The reason is that hypoxia and the consequent changes in the viscoelastic properties of SS-RBCs may play a role in the events of RBC adhesion and would add an unwanted level of complexity to this work whose focus is the β-AR and AT1R cross-regulation. In our experiments, we chose to test only RBCs that had the characteristic biconcave shape to avoid reticulocytes, which generally have irregular shapes as discussed in our previous work [23].

### Reagents

Human integrin αvβ3 protein (100 μg/mL, diluted in PBS) was purchased from Millipore (Billerica, MA). Alsever’s solution, epinephrine (16.39 nM, reconstituted in Alsever’s solution), valsartan (2 μg/mL, reconstituted in DMSO), and bovine serum albumin (BSA; 100 μg/mL, reconstituted in PBS) were purchased from Sigma-Aldrich (St. Louis, MO). RBCs were treated with the reagents at 37°C for 30 min. We note that the peak value of plasma valsartan concentration after a single 80–160 milligrams oral dose administration of clinical valsartan tablet was approximately 2 μg/mL [53, 54]. Also, the maximum daily valsartan dose for adults is 320 mg, and the peak plasma concentration was 5.5 μg/mL [55]. By testing the effects of valsartan at different concentrations on SS-RBC ICAM-4 expression using SMFS, we found that there was no significant difference between the effect of valsartan at 1, 2, and 10 μg/mL (the corresponding active ICAM-4 densities were 11.46 ± 1.18%, 8.77 ± 1.58%, and 7.32 ± 1.32%). Based on these results, which cover the entire spectrum of clinical valsartan dose administration, we decided to use 2 μg/mL in our experiment, since it is close to the peak plasma concentration resulted from the recommended starting dose.

### AFM probe functionalization

Silicon nitride AFM probes were purchased from Bruker Nano Inc. (Camarillo, CA). The tip height was 2.5–8 μm and the nominal tip radius was 20 nm. The nominal spring constant of the applied cantilever is 30 pN/nm. The actual spring constant under the experimental condition (in Alsever’s solution at 37°C) was computed using a thermal noise collecting method [56, 57]. The cantilevers were first soaked in 2% v/v 3-aminopropyltriethoxysilane (APTES) in acetone (Sigma-Aldrich, St. Louis, MO) for 10 min, then rinsed with deionized (DI) water. Cantilevers were treated with 0.5% v/v glutaraldehyde (solution in DI water) for 30 min to adhere the glutaraldehyde molecule linker, then rinsed again with DI water and incubated in 100 μg/mL αvβ3 solution to attach the ligand. After 30 min, the cantilevers were rinsed again with DI water and immersed in 100 μg/mL BSA for 1 min to block the residual aldehyde groups. Cantilevers were stored in PBS at 4°C and used within three days.

### AFM setup

**Fig 2:**
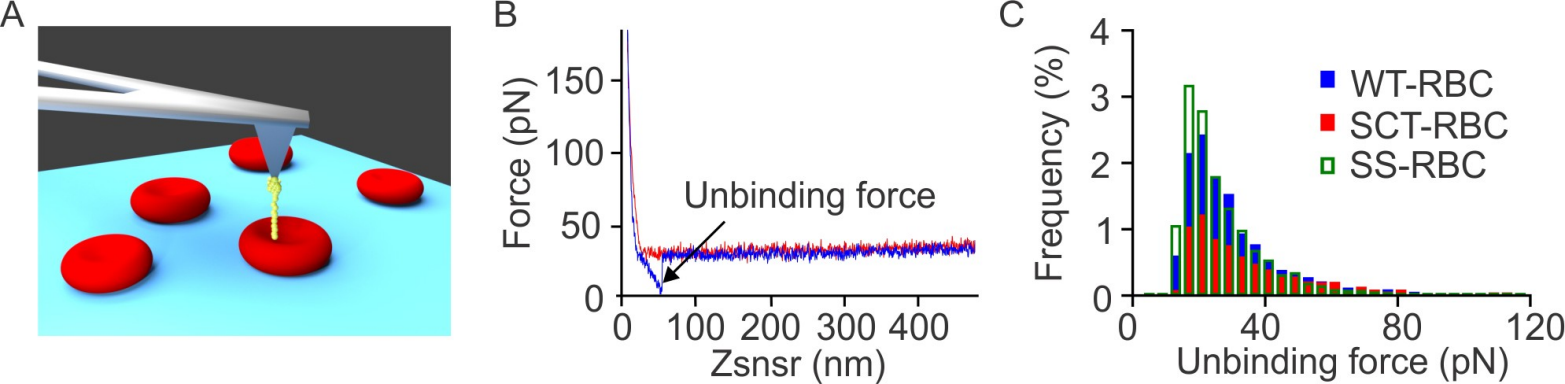
Schematic of a SMFS experiment along with a representative force-displacement measurement and the corresponding histogram. (A) AFM probing illustration. The yellow colored element represents the glutaraldehyde linker and the ligand αvβ3 protein molecule attached to the cantilever tip. (B) Representative force-displacement curve. The red curve denotes approach and the blue curve denotes retraction. (C) Frequency distributions of unbinding forces between ICAM-4 and αvβ3 in WT-, SCT-, and SS-RBCs.

All experiments were performed using the MFP-3D-BIO AFM (Asylum Research, Santa Barbara, CA), which was mounted on an inverted microscope (Zeiss Axiovert A1, Oberkochen, Germany). The microscope gave a clear image of RBCs, which were seeded in a transparent glass bottom petri dish allowing us to choose RBCs that were less than 10 μm in diameter and maintained a distinct circular biconcave shape. For each experimental condition, 5 RBCs were tested from each subject. A schematic of a functionalized AFM probe scanning the membrane of a RBC is shown in Fig 2A.

Force-distance curves were recorded at 32×32 points as the cantilever scanned over a 1μm^2^ area of the RBC surface. Each curve contained a full approach and retraction trace (as shown in Fig 2B). We have shown that 1 μm^2^ scanning area for SMFS experiments on RBCs gives a representative result for the specific tested RBC [6]. When unbinding events occurred between the αvβ3-functionalized cantilever and ICAM-4 receptors expressed on the RBC membrane, an unbinding force was displayed as the cantilever was retracted from the cell surface [8, 23]. The unbinding force was measured as the magnitude of the abrupt drop to zero of the retraction force-displacement curve (Fig 2B). All data were processed using our in-house developed code named FRAME (Force Review Automation Environment) [58].

The cantilever approach/retraction speed was held constant at 800 nm/s, which corresponds to a nominal loading rate of 24,000 pN/s. At this speed, the force measurements were not significantly affected by hydrodynamic drag (Fig 2B) as it is clear from the overlapping between the approach and retraction curves before and after contact with the RBC membrane [59]. We only considered the unbinding events for which the effective spring constants (K_eff_) were less than 10 pN/nm which, in combination with the cantilever moving speed, ensures that the influence of K_eff_ on the magnitude of the unbinding force for the specific loading rate is not significant for SMFS experiments [60]. The effective spring constants were calculated from the slopes of the curves adjacent to the unbinding force drops [61, 62]. A specific threshold was set to control the indentation depth and to apply the same nominal maximum force to each RBC. The probe traveled a distance of ~200 nm, which corresponded to a contact time between the probe and the RBC membrane of ~1/4 s before retraction.

To illustrate the population of the active ICAM-4 receptors on the RBC surface, the collective frequencies were showed in box-and-whisker plots. Collective frequency (CF, %) was defined as the percentage of all unbinding events with respect to the total number of measurements (32×32 = 1024 for each cell). The CF revealed the percentage of active ICAM-4 receptors that were able to bind to αvβ3. This approach allowed the observation of the modulation of the CF of activated ICAM-4 receptors in the presence of valsartan and the other biochemical reagents.

### Statistical methods

The unbinding forces between αvβ3 ligands and ICAM-4 receptors are reported using frequency (%) distribution which states the percentage of events whose corresponding unbinding forces were within each bin's width (Fig 2C). The CF results are reported as mean ± standard error (SE) and illustrated by box-and-whisker plots (GraphPad Prism, GraphPad Software, Inc., La Jolla, CA). Significance was determined using one-way analysis of variance (ANOVA). The results were considered significant if p<0.05.

## Results and discussion

First, we verified previous findings that epinephrine stimulates the activation of ICAM-4 receptors on WT-, SCT- and SS-RBCs [9, 23]. Epinephrine, as a catecholamine, acts on β-ARs and stimulates the cAMP-PKA-dependent signaling pathway. The purpose of these experiments was to exclude the variance due to different blood samples and only compare the variation of active ICAM-4 receptors caused by the different reagents that we used. We employed the SMFS assay to scan a 1 μm × 1 μm membrane areas of individual RBCs with an AFM cantilever functionalized with αvβ3 to detect variation of active ICAM-4 receptors and measure the unbinding forces between active ICAM-4 and αvβ3 under different experimental conditions.

**Fig 3:**
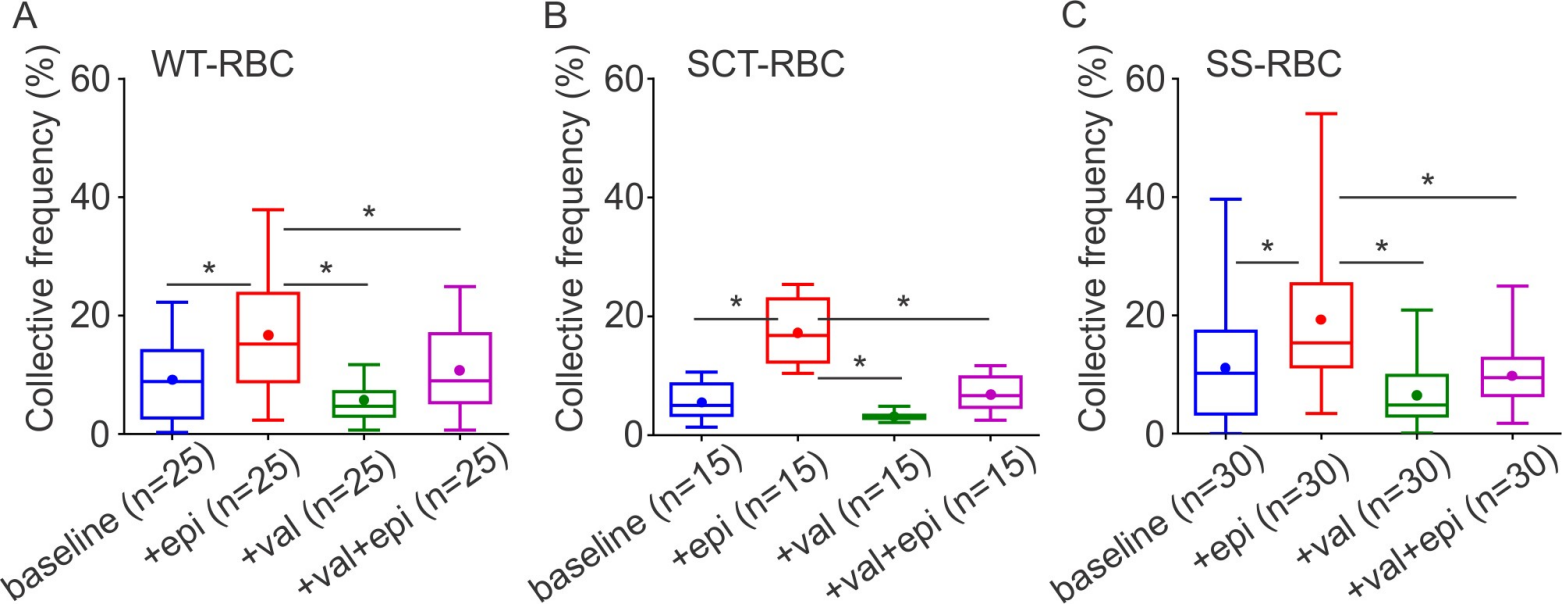
**Box-and-whisker plots of the CF of active ICAM-4 receptors measured on (A) WT-RBCs, (B) SCT-RBCs, (C) SS-RBCs.** Data are shown as the median with minimum and maximum whiskers, and the mean is denoted as a color dot. Significance between conditions is denoted as * such that *p*<0.05. The n on the x axis indicates the total number of mature RBCs tested in each group: WT-RBC: 5 subjects; SCT-RBC: 3 subjects; SS-RBC: 6 subjects.

Treatment with epinephrine caused a significant increase in the CF of active ICAM-4 receptors on the surface membrane of WT-RBCs from 8.98 ± 1.20% at baseline to 17.00 ± 1.89% (p = 0.0003; Fig 3A). The CF of active ICAM-4 receptors of SCT-RBCs was measured to be 5.58 ± 0.76% at baseline, which is similar to the CF obtained for WT-RBCs (p = 0.4696). Treatment of SCT-RBCs with epinephrine significantly increased the CF of active ICAM-4 receptors to 17.11 ± 1.50% (p<0.0001; Fig 3B). The CF of active ICAM-4 adhesion was 11.68 ± 1.68% for SS-RBCs at baseline, which is higher than that measured for WT- and SCT-RBCs, but not significantly (p = 0.9999, 0.1768). Similar to the observations made in WT- and SCT-RBCs, SS-RBCs treated with epinephrine demonstrated significantly higher CF of active ICAM-4 receptors 20.58 ± 2.76% (p = 0.0019; Fig 3C) compared to baseline.

### Valsartan suppresses the activation of ICAM-4 receptors induced by epinephrine

Valsartan is an AT1R blocker and a common clinical drug for treatment of hypertension and heart failure. In addition, it has been demonstrated that in cardiomyocytes, HEK 239 and COS-7 cells valsartan can simultaneously block downstream signaling of both AT1R and β-AR by affecting AT1R and β-AR heterodimers by diminishing the coupling between a β-AR and its corresponding Gs protein [36]. In this work, we investigated if treatment of RBCs with valsartan would affect the CF of active ICAM-4 receptors expressed on the surface membrane of the RBCs. We found that valsartan-treated WT-RBCs exhibited similar CF of active ICAM-4 to untreated WT-RBCs (5.20 ± 0.57% vs. 8.98 ± 1.20%, p = 0.2178; Fig 3A), but significantly lower CF of active ICAM-4 than epinephrine-treated WT-RBCs (5.20 ± 0.57% vs. 17.00 ± 1.89%, p<0.0001; Fig 3A). Then, we investigated if valsartan affects epinephrine stimulation of active ICAM-4 adhesion receptors. To this end, we sequentially incubated WT-RBCs with first valsartan and then epinephrine and compared their combined effect on the CF of ICAM-4 receptors with that of epinephrine exposure alone. We demonstrated that pre-treatment with valsartan resulted in a significant decrease in the expected epinephrine-induced activation of ICAM-4 adhesion receptors (10.41 ± 1.35% vs. 17.00 ± 1.89%, p = 0.0045, Fig 3A).

Similar experiments were performed on SCT-RBCs. As with WT-RBCs, SCT-RBCs showed no distinct change in the CF of active ICAM-4 when RBCs treated only with valsartan (3.13 ± 0.22% vs. 5.58 ± 0.76%, p = 0.2497, Fig 3B). However, when SCT-RBCs were treated with valsartan prior to their exposure to epinephrine, the CF of ICAM-4 receptors was significantly lower compared to treatment with only epinephrine (6.87 ± 0.91% vs. 17.11 ± 1.50%, p<0.0001, Fig 3B).

Similar experiments were then performed on SS-RBCs. Similarly to WT-RBCs and SCT-RBCs, SS-RBCs exhibited a comparable level of active ICAM-4 to baseline SS-RBCs with valsartan treatment alone (6.41 ± 0.91% vs. 11.68 ± 1.68%, p = 0.1195, Fig 3C), but showed a significant decrease with valsartan prior to epinephrine treatment compared to treatment with only epinephrine (10.07 ± 1.02% vs. 20.58 ± 2.76%, p = 0.0004, Fig 3C).

At baseline, WT-, SCT- and SS-RBCs demonstrated similar unbinding forces between the αvβ3 ligand and ICAM-4 (Fig 2C). In addition, neither epinephrine nor valsartan altered the unbinding force level between the αvβ3 ligand and ICAM-4 receptor, suggesting the reagents affect only the activation of ICAM-4 receptors, and not the strength of their interactions with αvβ3.

AT1R blockade significantly decreased the epinephrine-induced increase in the CF of active ICAM-4 adhesion receptors in WT-, SCT- and SS-RBCs. These findings suggest that valsartan efficiently suppressed the effect of epinephrine on the activation of ICAM-4 receptors in WT-, SCT-, and SS-RBCs. As postulated in previous research [36], β-AR and AT1R may form a constitutive complex on the RBC membrane. Administration of valsartan could act on the complex, not only blocking the action of AT1R but also uncoupling the β-AR and its cognate Gs protein. In this case, epinephrine could no longer effectively stimulate β-AR and the following cAMP-PKA-dependent pathway through Gsα dissociation. The nature of the uncoupling effect may involve a conformational change on β-AR making it no longer favorable to bind to Gs protein which is currently under investigation [36, 63].

## Conclusions

In this study, we explored the effect of valsartan on the activation of ICAM-4 receptors on WT-, SCT- and SS-RBCs. We observed that epinephrine significantly increased the surface percentage of active ICAM-4 receptors on WT-, SCT- and SS-RBCs. Importantly, we showed that although exposure to valsartan alone did not have a significant effect on basal level adhesion, pre-treatment of RBCs with valsartan prior to epinephrine stimulation resulted in a significant decrease in the percentage of active ICAM-4 receptors compared to treatment with only epinephrine. This may have major clinical implications as our data suggest that valsartan can modulate SS-RBCs adhesion, which is a known contributor to vaso-occlusion. Our findings suggest that administration of valsartan could mitigate the vaso-occlusive consequences of SCD and may open new avenues for the development of novel therapeutic targets.
